# Open-ComBind: harnessing unlabeled data for improved binding pose prediction

**DOI:** 10.1007/s10822-023-00544-y

**Published:** 2023-12-08

**Authors:** Andrew T. McNutt, David Ryan Koes

**Affiliations:** Department of Computational and Systems Biology, University of Pittsburgh, Pittsburgh, PA USA

**Keywords:** Molecular docking, Machine learning, Structure-based drug design, Open-source

## Abstract

Determination of the bound pose of a ligand is a critical first step in many in silico drug discovery tasks. Molecular docking is the main tool for the prediction of non-covalent binding of a protein and ligand system. Molecular docking pipelines often only utilize the information of one ligand binding to the protein despite the commonly held hypothesis that different ligands share binding interactions when bound to the same receptor. Here we describe Open-ComBind, an easy-to-use, open-source version of the ComBind molecular docking pipeline that leverages information from multiple ligands without known bound structures to enhance pose selection. We first create distributions of feature similarities between ligand pose pairs, comparing near-native poses with all sampled docked poses. These distributions capture the likelihood of observing similar features, such as hydrogen bonds or hydrophobic contacts, in different pose configurations. These similarity distributions are then combined with a per-ligand docking score to enhance overall pose selection by 5% and 4.5% for high-affinity and congeneric series helper ligands, respectively. Open-ComBind reduces the average RMSD of ligands in our benchmark dataset by 9.0%. We provide Open-ComBind as an easy-to-use command line and Python API to increase pose prediction performance at www.github.com/drewnutt/open_combind.

**Supplementary Information:**

The online version contains supplementary material available at 10.1007/s10822-023-00544-y.

## Introduction

Drug discovery is a complex, multifaceted process involving the identification of small molecules capable of interacting with specific biological targets to produce a therapeutic effect. Computer-aided drug design (CADD) tools allow for the reduction of the enormous complexity and cost of the traditional drug discovery pipeline [1]. CADD can be broadly divided into two main techniques: structure-based and ligand-based. Structure-based drug design (SBDD) uses the knowledge of the three-dimensional structure of a target protein to guide the selection of molecules that can bind with high affinity and selectivity [2, 3].

Molecular docking is a vital tool in the SBDD toolbox that predicts the non-covalent binding of two molecules in three-dimensional space. Knowledge of the ligand binding pose is critical for many downstream drug discovery tasks, such as lead optimization, affinity prediction, and virtual screening. Typical molecular docking algorithms either utilize energetics of interactions or the statistics of known binding interactions to predict the relative placement of the molecules [4]. Conventional docking algorithms, such as Autodock Vina [5] or Glide [6, 7], and even deep learning-based docking algorithms, like gnina [8]or DiffDock [9], predict the binding of every ligand separately even when multiple ligands are docked to the same receptor structure. Many drug targets are unsuitable for molecular docking due to a lack of suitable crystal structures (i.e. large ligand volume difference with cognate ligand or no available *holo* crystal structures). In these cases, alternative approaches must be employed.

One such approach is ligand-based drug design (LBDD), which relies on the analysis of known active compounds rather than the target protein itself. LBDD typically involves comparing the chemical features of known actives to those of putative inactives in order to identify pharmacophores - sets of chemical properties necessary for activity against a target [10]. These pharmacophores can be one, two, or three-dimensional, providing an understanding of active molecule properties or shape [11, 12]. The generated pharmacophores are used to screen large libraries of compounds to find drug-like molecules with similar properties. LBDD allows for fast screening of incredibly large molecule libraries. Still, its reliance on empirical observations means it can struggle to capture subtle differences between closely related analogs. The results may not always generalize to a new series of compounds.

There have been several recent works that integrate LBDD with molecular docking to improve predictive performance. [13] and [14] utilize LBDD techniques to determine protein conformations that will increase docking performance with a given drug molecule. [15] use protein-ligand interaction fingerprints to increase the scoring power of standard molecular docking scoring functions. [16] and [17] harness knowledge of known bound poses to guide the molecular docking of novel ligands binding to the same receptor. These methods are able to reduce the sampling space of the ligands during docking by using known bound ligand poses as a bias to their sampling procedure. [18] integrated structure-based docking from Glide, a commercial docking software, [6, 7] and 3D ligand similarities to increase pose prediction performance with their docking pipeline ComBind. Their work allows the use of abundant information on known active molecules that have no known bound structure to improve pose selection. Using Glide to dock a diverse protein-ligand benchmark set, they derive two distributions describing the pairwise pose similarity of poses near the ground truth and the pairwise pose similarity of all docked poses with respect to features such as hydrogen bonds or hydrophobic contacts. During the pose selection procedure, the feature distributions are converted to energy-like terms using the log ratio of the near-native similarity distribution to the all-pose similarity distribution. These likelihood terms are used in combination with Glide’s pose score to select a pose for a query ligand as well as for a set of ligands known to bind to the same receptor that do not require resolved structures. Their pipeline showed increased pose selection performance over only using Glide’s pose score, however, their pipeline utilized closed-source, licensed tools for pose generation and featurization, limiting its adoption by the molecular modeling community.

Here we present an easy-to-use, open-source alternative to ComBind that we call Open-ComBind. The open-source, deep learning-based molecular docking software gnina is used to generate high quality ligand poses, and open-source tools like ProDy [19, 20] and RDKit are used to featurize the docked compounds. Using a likelihood framework identical to ComBind, we utilize a set of helper ligands without known structural information to improve the pose quality of our docked ligand. The pose selection procedure strikes a balance between the individual pose scores assigned by the per-ligand deep learning scoring functions and the similarities and differences between the poses of various ligands. As a result, Open-ComBind produces a set of optimized poses for all ligands simultaneously, without requiring a shared ligand scaffold or knowledge of any ligand’s binding pose.

## Methods

Here, we detail the dataset used in the creation of the docking pipeline as well as the dataset employed for evaluation of our pipeline. We describe the creation of inter-pose similarity statistics by performing cross-docking on a diverse protein-ligand benchmark dataset. The Open-ComBind docking pipeline is described, harnessing the similarity statistics to performing pose selection on a ligand of interest, the ‘docking ligand’, using a set of ligands with unknown bound structures, the ‘helper ligands’. Finally, an experiment to determine the validity of Open-ComBind’s underlying hypothesis: ‘distinct ligands bind to a receptor in similar ways’.

### Data

#### Similarity dataset

Following [18], a list of 30 target proteins (Table S1) representative of all major families of drug targets is used to generate the pairwise pose feature similarity distributions. The entire benchmark dataset of 421 protein-ligand pairs is used to generate the similarity statistics.

#### Benchmark dataset

Generating the dataset used for evaluating pose prediction pipelines necessitates removal of easy-to-dock ligands. Following [18], we filter the Similarity Dataset to remove any ligand that has greater than 50% of its atoms in the maximum common substructure computed with the cognate ligand of the receptor structure used for docking. If a new ligand shares most of the same structure as the cognate ligand then its probable the pocket has an appropriate configuration to bind the similar ligand. This reduces the set to 245 protein-ligand complexes for determining the performance of a pose prediction pipeline. These ligands are used as the ‘docking ligand’ during the evaluation of the pipeline.

#### Helper ligands

Helper ligands are a set of ligands known to bind to the receptor of interest with $<1\mu M$ affinity, but may not have a solved bound structure. These ligands are used along with the ‘docking ligand’ in the Open-ComBind pipeline to improve pose selection of both the ‘docking ligand’ and the ‘helper ligands.’

We utilize the same two sets of helper ligands as in [18]: one in which the helper ligands do not share a scaffold with the docking ligand, termed ‘High-affinity helper ligands’, and the other in which the ligands have the largest Maximum Common Substructure (MCSS) with the docking ligand, termed ‘Congeneric series helper ligands’. We use the MCSS definition from [18] to ensure we are using the same set of helper ligands.

### Derivation of similarity distributions

Here we detail the development of pairwise similarity distributions for near-native poses and all docked poses using the Similarity Dataset.

#### Protein and ligand pre-processing

Protein and ligand pre-processing separates the protein and ligand into separate docking-ready structures. Initially, a set of co-crystallized protein-ligand structures for each receptor in the Similarity Dataset is downloaded from RCSB.org [21, 22]. The ligand of interest in each co-crystallized structure is then designated based on its residue name. Using ProDy [19, 20], separate files are generated for the protein, ligand, waters, and other heteroatoms that are present in the original crystal structure. The instance coordinates of the ligand structure are downloaded directly from RCSB.org [21, 22] using their File Download Service to ensure proper coordinate and bond information. Using only the protein and ligand complex, we align all structures of a receptor based on the residues closest to the binding pocket. Protein residues within 15 Å of the ligand are used to align each receptor structure with PyMol [23] to the alphabetically lowest PDB ID (PDB IDs specified in Table S1). The co-crystallized ligand’s coordinates are transformed according to the receptor alignment object. The aligned ligand and receptor are then separated into their own files to provide a ground-truth ligand and a *holo* structure for cross-docking with non-cognate ligands. After removal of the ligand, missing residues, atoms, and hydrogens are added (at a pH of 7.0) to the receptor structures using PDBFixer and minimized, ensuring interactions are detected properly after docking. Missing atoms and hydrogens are minimized using the default OpenMM forcefield. Finally, a gnina docking command template is created for each protein. The gnina docking command template specifies the docking protein structure (i.e. the aligned-to receptor structure) as well as the binding box location, defined by the cognate ligand of the docking receptor.

Docking with gnina requires knowledge of the 3D structure of the ligands to be docked. Conformations are generated for ligands using Experimental-Torsion with basic Knowledge Distance Geometry (ETKDG) [24, 25] to yield likely conformations of the ligand based on trends seen in crystal conformations. Using RDKit, we generate 50 conformations, and use the Universal Force Field (UFF) to minimize and report the energy of each generated conformation. Combining the ten lowest energy conformations into a single file for each ligand. A set of low-energy conformations gives a higher likelihood that one of the conformations is close to the ground-truth ligand conformation. Additionally, gnina docking keeps rigid bond angles and lengths, so using multiple conformations of a ligand during docking allows for exploration of different bond angles and lengths.

#### Cross-docking

After both ligand and protein preparation, cross-docking is performed with gnina. All of the ligands are docked against the receptor structure used for the alignment, the alphabetically lowest PDB ID (Table S1). The receptor’s cognate ligand identifies the binding site. Docking is performed using all default parameters, except for exhaustiveness, min_rmsd_filter, and num_modes (Table S2). exhaustiveness is increased to 16 to perform more sampling of the ligand during docking. min_rmsd_filter is reduced to 0.01 to allow highly similar, high-scoring poses to be output. num_modes is set to 30 to ensure we have a max number of 300 total poses for each ligand if we have ten starting conformations for each ligand. Following the docked pose statistics of [18], which have up to 300 poses per docked ligand, we attempt to sample 300 docked poses per ligand complex. Additionally, gnina tends to produce distinct poses in comparison to Glide which tends to output highly similar poses, often leading to generating several near-native poses. Therefore, we utilize several ligand conformations during docking to increase the likelihood that gnina outputs more than one pose close to the native state. After docking, we re-sort the combined output poses from gnina for each ligand according to the ‘CNNscore’, a score in the range [0,1] denoting the likelihood a ligand pose is correct [26]. Following [18] and to reduce computation, only the top 100 docked poses of each ligand are used for subsequent steps.

#### Featurization

The top 100 docked poses of each ligand are investigated for their interactions with the receptor and their distance from the ground truth, if available. Each pose has associated docking scores from both Autodock Vina and the Convolutional Neural Network (CNN) scoring functions in gnina. Additionally, the interaction fingerprint of each docked pose is computed. The interaction fingerprint profiles and scores the hydrogen bonds, salt bridges, and hydrophobic contacts between the protein and ligand. Hydrogen bond scores are a product of the distance between the donor and the acceptor’s hydrogen and the angle between the donor, hydrogen, and acceptor. However, since gnina ignores hydrogens in both the input and output, we add hydrogens to the docked poses with RDKit and minimize the hydrogen atom placement with UFF, ignoring the effect of the protein environment. Both the salt bridge and hydrophobic contact scores are simply the distance between the involved atoms. Unique to Open-ComBind, salt bridges are determined via ligand substructure matches to SMARTS strings, taken from Pharmit [27], for the detection of positive and negative ions rather than using atoms with a formal charge since gnina does not modify the formal charge of the input molecule based on the receptor environment. These scores are summed by protein residue for each feature type.

Following the featurization of individual poses, we can calculate the similarity of pairs of poses based on their features. A pseudo-Tanimoto Similarity is used for the similarity of hydrogen bonds, salt bridges, and hydrophobic contacts. Since the features are not bit vectors, the Tanimoto Similarity between pose *i* and *j* is calculated as follows:

$$\begin{aligned} \mathrm {psuedo-}TS_{ij} = \frac{1+\sum \limits _{r\in {\mathcal {P}}} \sqrt{f^r_i} \sqrt{f^r_j}}{2 + \sum f_i + \sum f_j + \sum \limits _{r\in {\mathcal {P}}} \sqrt{f^r_i} \sqrt{f^r_j}} \end{aligned}$$

where *f* is one of the features and ${\mathcal {P}}$ are all of the residues of the docking protein. An additional inter-pose feature is calculated via the RMSD between the MCSS of the two poses. We utilize a set of MCSS parameters that find a substructure with both matching atom IDs and bond orders. However, halogen atoms are allowed to match any other halogen atoms and rings are constrained to only match other, complete rings. We do not consider the MCSS if the number of heavy atoms in the MCSS is less than half the number of heavy atoms in the smallest ligand.

#### Generating similarity statistics

Given our inter-pose features, we can now compute the extent to which ligands binding to the same receptor do so in a similar manner. We first define the ‘native distribution’, which looks at the inter-pose similarity between only poses with < 2Å RMSD from the ground truth, denoted as $f(s(\cdot ,\cdot )|\,\text {Native})$. We next define the ‘reference distribution’ to be the inter-pose similarity between all pairs of the top 100 poses output by gnina, denoted as $f(s(\cdot ,\cdot )|\,\text {Reference})$. Both distributions are determined via a Gaussian kernel density estimate on each of the inter-pose features. Following [18], the Gaussian kernel density estimate uses a standard deviation of 0.03 for interaction similarities and 0.18 for MCSS RMSD. Reflected boundaries are used to reduce bias near the boundaries.

The distributions for MCSS RMSD were both capped at 6 Å to eliminate the effect of the sparse distribution for higher RMSDs. Any values greater than 6 Å were set to 6 Å.

### Open-ComBind pose prediction pipeline

**Fig. 1:**
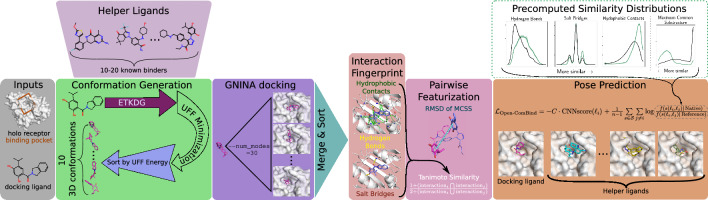
Open-ComBind pose prediction pipeline: the user provides a docking ligand, a *holo* receptor structure, and a defined binding pocket on the structure. The user can then define a set of helper ligands: molecules with $<1\mu M$ affinity to the receptor but not requiring any bound structure. A set of conformations is generated for the docking ligand and the helper ligands. gnina is used to dock the docking and helper ligands, outputs are sorted by CNNscore. The poses for each ligand are featurized and inter-pose similarities are calculated between all pairs of poses. Finally, the poses for the docking ligand as well as the helper ligands are selected using the Open-ComBind objective function which harnesses the pre-computed similiarity distributions

Open-ComBind provides a pipeline (Fig. 1) that prepares protein and ligand structures for docking, a suite of methods to compute the similarity of features from pairs of docked ligands, as well as predicting a set of poses utilizing pre-computed similarity distributions. Protein-ligand ground-truth complexes, usually determined via X-ray crystallography, are manipulated to extract separate protein and ligand structures after alignment of the pocket residues of similar protein structures. Ligands are prepared for docking by generating a set of low-energy 3D ligand conformations. Docking is run on a single receptor conformation with all of the generated conformers per ligand and the output for each ligand is sorted according to its docking score. Featurization of the top poses for each ligand includes their docking score and fingerprint of the protein-ligand interactions. Feature similarities are computed between all pairs of ligand poses, both inter- and intra-ligand pairs, utilizing the pose interaction fingerprints and the RMSD between the MCSS of the pose pair. We use the same protein and ligand pre-processing, conformation generation, docking, and featurization procedure as enumerated in “Derivation of similarity distributions” section. Finally, a pose is selected for each docked ligand by maximizing the sum of the similarity between the pose selected for each ligand and the pose’s docking score.

Poses are selected for the docking ligand and the set of helper ligands utilizing the inter-pose features in tandem with the pre-computed native and reference distributions. Adhering to the same procedure as [18], we randomly initialize pose selections and randomly iterate through the ligands, picking a pose that maximizes the objective:

$$\begin{aligned}{} & {} {\mathcal {L}}_{\text {Open-ComBind}} = -C\cdot \text {CNNscore}(\ell _i) \\{} & {} \quad + \frac{1}{n-1}\sum \limits _{s\in {\mathcal {S}}}\sum \limits _{j\ne i}\log \frac{f(s(\ell _i,\ell _j)|\,\text {Native})}{f(s(\ell _i,\ell _j)|\,\text {Reference})} \end{aligned}$$

where $\ell _i$ is the pose selected for the current ligand, ${\mathcal {S}}$ is our set of similarities, $f(s(\cdot ,\cdot )|\,\text {Native})$ and $f(s(\cdot ,\cdot )|\,\text {Reference})$ refer to the pre-computed native and reference similarity distributions, respectively, and *C* is a hyperparameter to weight the CNNscore. We attempt to identify the set of selected poses that optimizes this objective function through a greedy iterative process. We continue iterating through the ligands, updating the selected pose, until no new poses are selected for any ligands. This procedure is run 500 times to increase the likelihood of finding the global minimum. The poses selected by the objective function for each ligand are returned.

#### Selection of CNNscore weight

We select the hyperparameter, *C*, for the weight of the CNNscore (the pose score output by gnina [26]) within the Open-ComBind score function in the same way as [18]. The same cross-docked top 100 poses for each ligand used for computing the similarity statistics are pooled and sorted according to their CNNscore. For each consecutive cluster of 100 poses, we calculate the average CNNscore and the negative log-likelihood of those 100 poses being a correct pose (i.e. $\le 2$Å from the ground truth). A line is fit to the data and the slope is used as the weight for the CNNscore.

### Benchmark dataset evaluation

Cross-docking is the main focus of many docking studies as it most closely emulates docking experiments in drug discovery campaigns. We test the performance of Open-ComBind by performing cross-docking on the Benchmark Dataset. We utilize the protein and ligand pre-processing available in the Open-ComBind pipeline to prepare the ligands for cross-docking. A protein and ligand file are created for each complex of the Benchmark Dataset, however, binding site cofactors are manually added back to the protein, following ComBind’s preparation procedure for a fair comparison. Next, we create two sets of helper ligands for each ligand we are docking, as defined above: high-affinity binders and congeneric series. We then use the Open-ComBind Pose Prediction Pipeline (Fig. 1) on each ligand in which we are performing cross-docking with each set of helper ligands. When predicting poses for docking ligands of a given receptor, we omit that receptor’s docked ligand similarity statistics from the pre-computed similarity statistics used in the Open-ComBind objective function.

We run the evaluation, for each docking ligand, using five different random seeds to determine the variability of Open-ComBind given slightly different ligand poses. The random seed are used for the creation of the conformations of the docking ligand and helper ligands as well as the gnina docking procedure.

### Hypothesis evaluation

[18] built ComBind on the hypothesis that distinct ligands bind to a receptor in similar ways. We aim to investigate whether this underlying hypothesis of the pipeline is correct by comparing the value of the Open-ComBind objective function for the lowest RMSD ligand poses to the value of the objective when selecting from all gnina generated poses. We only look at target proteins in the Benchmark dataset containing at least ten ligands with a gnina generated near-native pose. In the following experiment, we only use ligands with a defined ground truth pose (i.e. the set of docking ligands for each target receptor in the benchmark dataset) and only ligands that have a gnina generated pose < 2Å RMSD from the ground truth. We first measure the value of the Open-ComBind objective function when we restrict our selection to the best gnina generated pose, according to lowest RMSD to the ground truth. We next evaluate the value of the Open-ComBind objective function when we select from all gnina generated poses of the ligands. If the underlying hypothesis of Open-ComBind is true, then the Open-ComBind objective function value when selecting from the best poses will be greater than or equal to the objective function value when selecting from all gnina generated poses. The Open-ComBind objective is optimized in a stochastic manner, so it is possible we will not converge on the globally optimal pose selections which is assumed to be the set of poses with the lowest RMSD to the ground truth.

## Results

### Similarity distributions

**Fig. 2:**
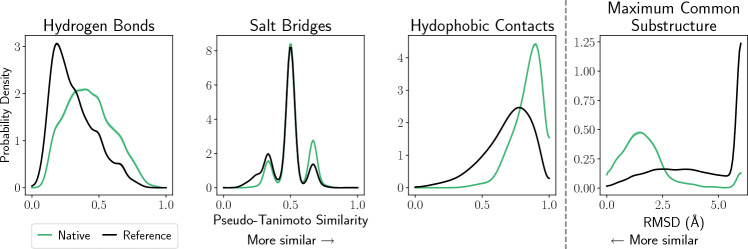
Pairwise similarities of different intra-molecular features and inter-pose similarities. The mean distribution across five seeds is plotted with the shading denoting the standard deviation

The pairwise feature similarity distributions are pivotal to the Open-ComBind objective function. Shifts from the reference to native distribution help to ensure the poses selected by the Open-ComBind objective are more likely to be pulled from the set of near-native poses. We calculate the pairwise feature similarity distributions by cross-docking all ligands in the Similarity Dataset using gnina. Then the top 100 poses of each ligand were analyzed for their interaction with the receptor structure used for docking. We see in Fig. 2 that all of the intermolecular interactions: Hydrogen bonds, Salt Bridges, and Hydrophobic contacts have higher similarity in the native distribution than in the reference distribution. The hydrogen bonds reference distribution is heavily right-skewed, while the native distribution is centered around 0.43 with a Gaussian-like appearance. Salt Bridges are much less frequent interactions, therefore both the native and reference distributions are heavily focused on the points $\frac{1}{3}$, $\frac{1}{2}$, and $\frac{2}{3}$ indicating that most ligands only make a small number of salt bridges with the protein. The native distribution shift is not as pronounced in the salt bridge due to its infrequency; however, we still see an increase in the density centered around $\frac{2}{3}$ and a slight decrease in the density around $\frac{1}{3}$ Pseudo-Tanimoto similarity relative to the reference distribution. Hydrophobic contact similarity is left skewed in both the native and reference distributions. The native distribution has a mean of 0.83, while the reference distribution has a mean of 0.69. The RMSD of the MCSS shows the same trend as the intermolecular interactions, with the native poses being more similar than the similarity distribution of all poses. Due to the cutoff at 6 Å we see a spike in both distributions at 6 Å. The spike in the reference distribution far outweighs the left tail.

The hyperparameter *C* is set to 1 following the slopes of the best fit lines in Figure S1.

### Docking results

**Fig. 3:**
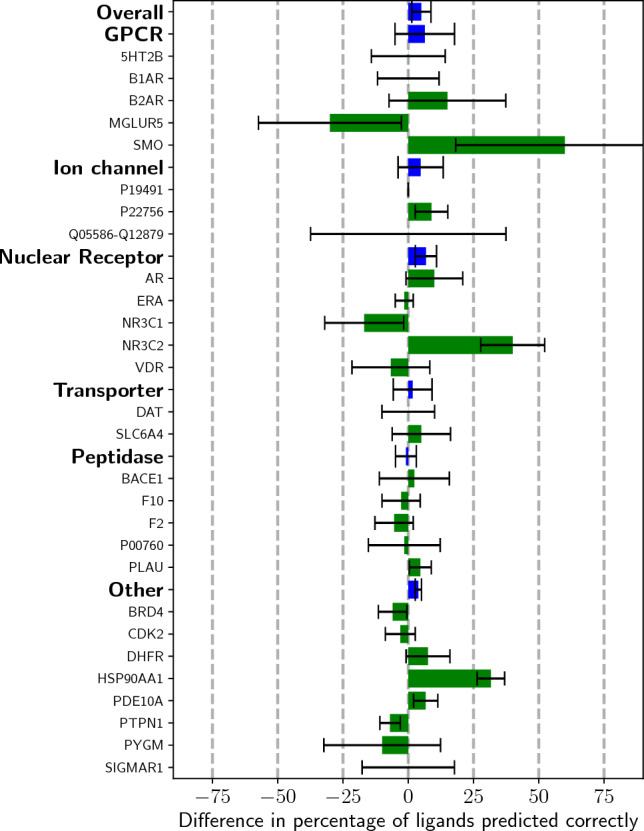
Average difference in percent of ligands whose pose is predicted correctly between gnina’s top scoring pose and the pose selected by Open-ComBind when high-affinity helper ligands are used. Error bars show the standard deviation across the five seeds. Performance with the congeneric series helper ligands is shown in Figure S2

After defining our pairwise similarity distributions and the hyperparameter for the weight of the CNNscore, we can utilize the Open-ComBind framework to select poses from gnina’s sampled poses. Binders lacking structural information are used as helper ligands to select docked poses for our ligand of interest that interact with the receptor in similar ways to the helper ligands. Utilizing the pairwise similarity distributions shown in Fig. 2 along with the gnina docking scores enables selection of near-native poses more often than when only the gnina score is used to select a pose (Fig. 3 and S2). Following [18], we compute the “Overall" statistic, which is the weighted average of the different protein classes according to the proportion of FDA-approved drugs for that class of proteins. Open-ComBind increases the Overall percentage of ligands with a correct pose by 5% and 4.5% for high-affinity and congeneric series helper ligands, respectively. Nuclear receptors, GPCRs, and ion channels have the greatest increase in performance for high-affinity ligands over only the gnina docking score, 6.8%, 6.3%, and 4.7%, respectively (4.7%, 3.2%, and 12.7%, respectively for congeneric series ligands). However, in contrast to [18], we find that many proteins show a reduced performance when utilizing the Open-ComBind framework relative to gnina. Additionally, for most of the proteins, we see large variances in Open-ComBind’s performance with different runs of gnina.

**Fig. 4:**
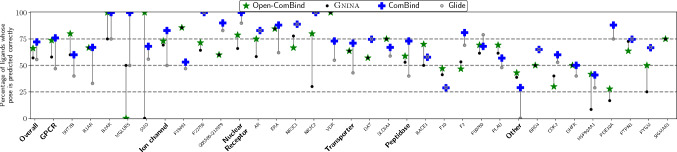
Comparing the docking performance of the best performing Open-ComBind seed and its associated gnina run to the performance of ComBind and Glide. Performance of ComBind and Glide scraped from [18] using WebPlotDigitizer [28]

When we compare our overall performance to that of [18] in Fig. 4 and S3, we see that our open-source version is slightly below the performance of ComBind. However, when comparing ComBind to Open-ComBind on each individual protein, we see a different story. ComBind tends to either increase or not change the percentage of ligands whose pose is predicted correctly across all of the proteins in the benchmark dataset. Open-ComBind has widely varying performance across different protein targets, with some protein targets seeing a major boost in performance for their docked ligands, like SMO and NR3C2 with a 100% and 50% increase, respectively. Other protein targets show a drastic decrease in the percentage of ligands whose pose is predicted correctly, for instance, MGLUR5 and NR3C1 with a 50% and 11% decrease, respectively. ComBind only has worse pose selection than Glide on one protein target when using high-affinity helper ligands, while Open-ComBind has worse pose selection than gnina on five protein targets across varying protein classes. This is all in spite of the fact that gnina and Glide demonstrate similar pose selection performance across a majority of the protein targets.

The RMSD to ground truth of poses selected by gnina is higher than the RMSD of the poses selected by Open-ComBind for the exact same ligand (Figures S4 and S5), average RMSDs of 2.67 and 2.43 Å for gnina and Open-ComBind using high-affinity ligands, respectively. Across all of the random seeds, there is a reduction in the average RMSD of the ligands when using Open-ComBind. However, Open-ComBind does not always choose a lower or equivalent RMSD pose for each ligand. This could be due to the fact that some of the featurizations do not contribute to better pose selections.

**Fig. 5:**
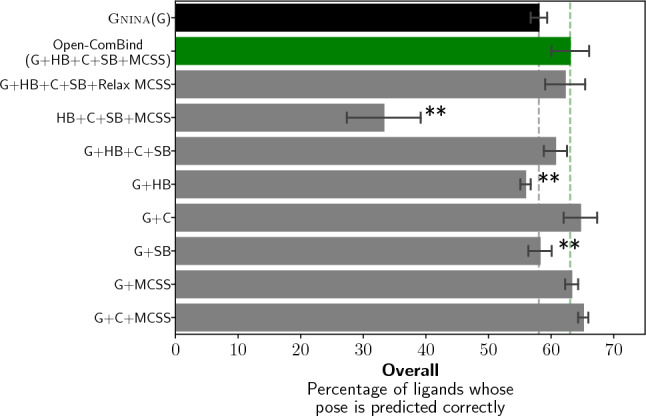
Removing components of Open-ComBind to determine their importance to the selection of docked poses when using the high-affinity helper ligands. ‘G’ indicates that gnina scores are used, ‘HB’,‘C’, and ‘SB’ indicate that hydrogen-bond, hydrophobic contact, and salt bridge similarity is used, respectively. ‘MCSS’ indicates that the RMSD between the MCSS of ligand poses is used and ‘Relax MCSS’ indicates a relaxed MCSS strategy was used where strict atom and bond typing were not enforced. ‘$$\cdot$$’ indicates a p-value less than 0.05 while ‘$$**$$’ denotes a p-value less than 0.01 in comparison to standard Open-ComBind

The importance of Open-ComBind docking components to the pose selection is inspected by removing pieces from the full docking pipeline to see their impact on performance. We see from Figs. 5 and S6 that removing the CNNscore from the pose selection process completely destroys Open-ComBind’s ability to select correct poses, dropping from 63.1 to 33.3% when using high-affinity helper ligands (62.5–38.8% when using congeneric series helper ligands). Removing individual features from Open-ComBind does not have as great an effect as removing the CNNscore, but can significantly impact Open-ComBind’s pose selection. The interaction fingerprint is pivotal to pose selection. Still, we see that only using the H-bonds (55.9%) or salt bridges (58.2%) gets about the same performance as gnina-only pose selection (58.1%). Using only Hydrophobic contacts in Open-ComBind is not significantly different (p = 0.20 and p = 0.29 for high-affinity and congeneric series helper ligands, respectively) from using all of the features. Removing the whole interaction fingerprint and only using the maximum common substructure RMSD between ligand poses slightly increases performance (63.3%, p = 0.44 and 64.6%, p = 0.11 for high-affinity and congeneric series helper ligands, respectively) but still shows improvements over gnina pose selection. Removing both the H-bonds and salt bridges from the Open-ComBind pose selection process gets about the same performance as when they are used (65.1%, p = 0.09 and 63.1%, p = 0.38 for high-affinity and congeneric series helper ligands, respectively), further demonstrating the inadequacy of H-Bond and salt bridges for pose selection improvements in Open-ComBind.

**Fig. 6:**
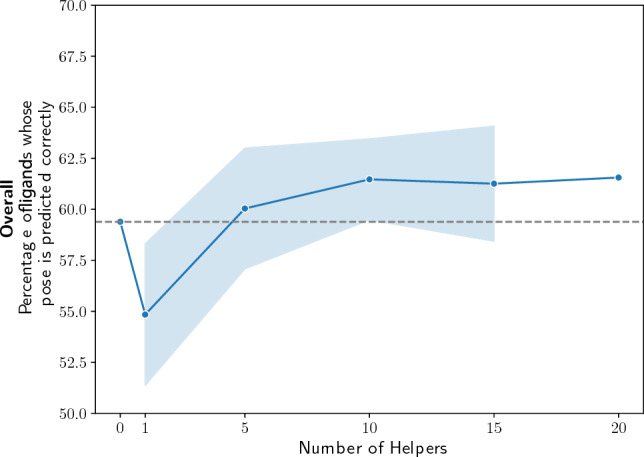
Reducing the number of similar helper ligands used in the Open-ComBind pipeline decreases the number of docked ligands whose pose is predicted correctly. The line is the average and the shaded area is the standard deviation across 15 different subsamples of helper ligands for each number of helper ligands

Pose selection performance should be dependent on the number of helper ligands we use during pose selection. When no helper ligands are used during the pose selection process, we see that the percentage of ligands whose pose is predicted correctly drops (‘gnina(G)’ in Fig. 5 and S6). However, we see in Figs. 6 and S7 that performance plateaus as more helper ligands are added. The performance with ten helper ligands, (61.47% and 62.55% for high-affinity and congeneric series helper ligands, respectively) is about the same as using all 20 helper ligands (61.56% and 62.59% for high-affinity and congeneric series helper ligands, respectively).

### Hypothesis evaluation

**Fig. 7:**
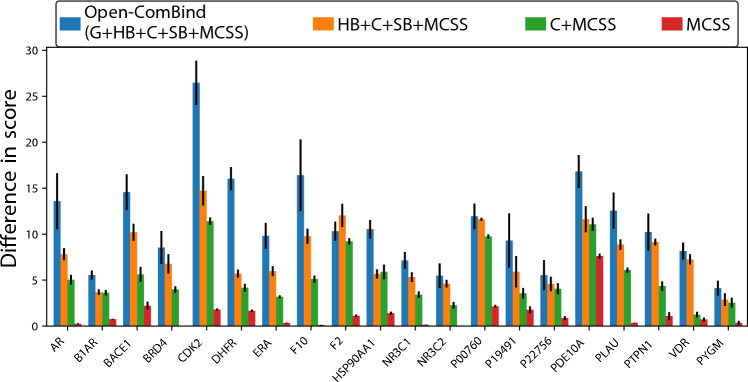
The difference of the Open-ComBind objective function when selecting from all gnina generated poses and the lowest RMSD to ground truth gnina pose. ‘G’ indicates that gnina scores are used, ‘HB’,‘C’, and ‘SB’ indicate that hydrogen-bond, hydrophobic contact, and salt bridge similarity is used, respectively. ‘MCSS’ indicates that the RMSD between the MCSS of ligand poses is used

The Open-ComBind objective function is formulated to encourage all the ligands being docked to assume similar interactions with the receptor. However, when we compare the Open-ComBind objective function value of the best gnina poses for each ligand to the optimal value when selecting from all gnina poses, we find that they are never the same when the full objective is used. (Fig. 7). The value of the objective function when only using the lowest RMSD to the ground truth pose found by gnina for each of the ligands being docked is less than selecting from all gnina poses, except when only the MCSS featurization is used. This implies that the underlying hypothesis of Open-ComBind is not necessarily true for these protein-ligand complexes during cross-docking. When we remove the hydrogen-bond and salt bridge interactions from the Open-ComBind objective function, the values from the two sets of poses come closer together. This is exacerbated when all interaction fingerprint features are removed from the Open-ComBind objective function and only RMSD of the MCSS is used and some proteins even have equal values of the objective function for both sets. The collection of poses identified by Open-ComBind is more similar in their interactions according to the objective function than the ensemble of lowest RMSD to ground truth poses. We additionally find that the Open-ComBind objective will not always select the native-pose when it is included in the list of poses to select from (Figure S8).

## Discussion

### Implementing an open-source ComBind alternative

Open-ComBind is an open-source alternative to ComBind, developed by [18], that has comparable increases in pose prediction performance over the respective baseline docking method. Building out Open-ComBind took a concerted effort as the original pipeline uses opaque software tools with unspecified methods for pose generation and featurization of the docked poses. The feature distributions generated with only the poses of a single ligand conformation gnina docking run did not show more similarity in the native distribution than in the reference distributions. By comparing the inter-pose RMSD pose of both gnina and Glide, we found that Glide was much more likely to output a set of highly similar poses while gnina did not. Therefore, we were able to increase similarity of the native distributions by docking a set of ten ligand conformations and combining the docking runs of each conformation into one single set of poses which were sorted by CNNscore. This produces a list of 100 conformations that has a higher likelihood of being less than 2 Å RMSD from the ground truth and reduces the RMSD between better scoring poses.

Differences between gnina’s and Glide’s output poses required further development of the featurization pipeline as Glide produces poses with hydrogens placed to maximize hydrogen bonding and alters the tautomeric form of the ligand to form salt bridges with the protein. We overcame these differences by adding hydrogens with RDKit during featurization and detecting salt bridge forming moieties with SMARTS string patterns. Placing the hydrogens in this manner ignores the location of hydrogen bond-forming donors and acceptors on the receptor; this likely decreases the strength of the hydrogen bonds seen in Open-ComBind in comparison to ComBind. We see that the Hydrogen bond similarities do not add information over only utilizing the CNNscore in Figs. 5 and S6, despite the fact that there are many hydrogen bonds detected in the benchmark dataset (Table S3). The usage of SMARTS string patterns for detection of salt bridges may also decrease the information provided by salt bridge similarity for increasing pose selection performance, but it is difficult to be sure as salt bridges are a rare occurrence in the benchmark dataset (Table S3).

The RMSD calculation between the MCSS of two ligand poses is the largest difference between Open-ComBind and ComBind. ComBind uses a MCSS strategy in which any two atoms can match in the graph regardless of atom type. We tested this relaxed MCSS strategy with Open-ComBind and found reduced performance (Figs. 5 and S6). Therefore, our MCSS calculation enforces stringent atom type matching (excluding halogens which can all match each other). We additionally require that bond orders be identical for the bond to be included in the MCSS and that rings can only match other complete rings. These MCSS requirements enforce a stronger match between substructures of ligands and ensure that the scaffold of a congeneric series is placed in a similar location for all ligands.

### Open ComBind performance

The exact poses selected by Open-ComBind vary significantly when the generated, docked ligand poses are changed via different random seeds. However, on average Open-ComBind tends to improve pose selection in comparison to the gnina baseline. We see in Figs. 4 and S3 that the standard deviation for a given protein is often much larger than its mean, indicating the results of Open-ComBind pose selection are highly dependent on the poses of the ligands. Altering the random seed affects the poses in the top scoring 100 used for the pose selection process, which can affect the consensus pose found for the docking ligand and helper ligands. We do see that the average RMSD decreases in comparison to the baseline docking method, gnina, regardless of the random seed used (Figures S4 and S5).

Hydrophobic contact similarity and MCSS RMSD are sufficient features to increase the pose prediction performance to that of the full Open-ComBind pipeline. This is likely due to the fact that hydrophobic contacts are the most common intramolecular interaction seen in the benchmark dataset by a factor of 6 (Table S3). Only utilizing these two features significantly decreases the computational cost of Open-ComBind. In fact, with congeneric series, we see (Figure S6) that only MCSS is required for the same performance as the full Open-ComBind pipeline. We expect congeneric series to have a much higher consistency in the placement of the MCSS because the MCSS is likely referring to the scaffold of the congeneric series. We also see that only 10 helper ligands are required to reach the pose prediction power of 20 helper ligands (Figs. 6 and S7). Reducing the number of helper ligands will also decrease the computational cost of the docking pipeline as a similarity matrix must be constructed for each feature which is ${\mathcal {O}}(N^2)$ with the number of ligands used, *N*.

[18] built the ComBind pose selection pipeline on the hypothesis that distinct ligands bind to the same receptor in similar ways. However, we show in Fig. 7 that the Open-ComBind objective function does not always select ligand poses that are the lowest RMSD to the ground truth. All of the proteins tested show a lower full objective function value when evaluating on the lowest RMSD near-native poses sampled by gnina in comparison to the objective function value when selecting from all sampled poses. However, we do see that our inspection of the underlying hypothesis agrees with the ablation studies in that the usage of only the hydrophobic contacts similarity and RMSD of MCSS improves the objective function’s performance in selecting near-native poses. Further, we see that use of only the RMSD of MCSS is able to find poses with the same objective function value as the lowest RMSD to ground truth poses. This implies that the RMSD of MCSS featurization aligns well with the underlying hypothesis, while the other features do not. It is likely that the hydrogen bonds, salt bridges, and hydrophobic contacts of the lowest RMSD to ground truth poses do not show as great of a consensus due to side chain rearrangement during binding of different ligands. Therefore, allowing side chain rearrangement during docking would likely improve the performance of Open-ComBind pose selection.

### Future directions

Open-ComBind is an easy-to-use, open-source alternative to ComBind, although more work is needed to match the performance gains achieved by [18]. Open-ComBind provides the molecular modeling community the opportunity to quickly and easily modify the featurization pipeline for increased pose prediction performance utilizing the large quantity of available, non-structural data. Features such as $\pi -\pi$ bonds and ligand shape similarity can be added easily to the docking pipeline. Since Open-ComBind uses gnina as the pose generator, we can utilize the internal representation of the CNN scoring functions within the docking pipeline as a featurization of the protein-ligand interaction for little extra computational cost. gnina also allows flexible sidechains during docking which could be used in the Open-ComBind framework for finding different pocket configurations to fit ligands of different sizes or ligands forming non-bonded interactions in different relative locations. Finally, Open-ComBind could be extended to predict the protonation state of the ligand to form better hydrogen bonds with the receptor and select better ligand poses than docking with the input protonation state.

### Ease to use API

Open-ComBind has been developed as a Python package with an accompanying command-line interface for running the entire pipeline. We provide the pre-processing, docking, featurization, and pose selection as easy-to-use Python modules or a single command on the command line. After installation of Open-ComBind and its pre-requisites from our github repository: www.github.com/drewnutt/open_combind, users can run the entire Open-ComBind pipeline with the command: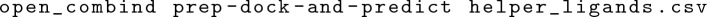 where helper_ligands.csv is a comma-delimited file of the helper ligands SMILES strings and identifiers. The user must specify the location of the raw protein-ligand complex to use for docking. Other protein-ligand crystals can be placed in the same directory for pre-processing.

Additionally, we provide scripts and instructions for using the Open-ComBind pose prediction pipeline with other molecular docking tools. Use of molecular docking tools that directly consider hydrogen bonds and predict ligand hydrogen coordinates would likely improve the utility of the hydrogen bond similarity in enhancing pose prediction.

## Conclusion

In this work, we developed Open-ComBind, an open-source alternative to ComBind, to increase pose prediction performance over gnina. Open-ComBind reduces the average RMSD to ground truth from gnina’s 3.24 Å to 3.03 Å for all ligands. We utilize ligands lacking structural information as well as a distribution of ligand feature similarity of both native poses and all poses. Open-ComBind utilizes pairwise similarities between sets of ligand poses to determine the poses which maximize inter-ligand similarity of binding. We see pose selection improvement equal to the full pipeline when using only hydrophobic contact similarity and the RMSD between the MCSS of ligand pairs. Additionally, we observe that Open-ComBind only requires ten helper ligands to reach the pose prediction performance of using 20 helper ligands. We provide the Open-ComBind in an easy-to-use API as well as source code and data to use the Open-ComBind pipeline at our GitHub repo: www.github.com/drewnutt/open_combind/

## Supplementary Information

Below is the link to the electronic supplementary material.

Supplementary file1 (PDF 512 kb)

## Data Availability

Information for downloading the datasets are available at www.github.com/drewnutt/open_combind/.

## Code availability

Our pipeline is open source and available under a CC BY-NC-SA 3.0 license. The full Open-ComBind pipeline code, documentation, and evaluation scripts are available at www.github.com/drewnutt/open_combind/.

## References

[1] Sliwoski G, Kothiwale S, Meiler J, Lowe EW (2014) Computational methods in drug discovery. Pharmacol Rev 66(1):334–395 24381236 10.1124/pr.112.007336 PMC3880464

[2] Gubernator K, Böhm H-J, Mannhold R, Kubinyi H, Timmerman H (1998) Structure-based ligand design. Wiley Online Library, New York

[3] Anderson AC (2003) The process of structure-based drug design. Chem Biol 10(9):787–797 14522049 10.1016/j.chembiol.2003.09.002

[4] Kitchen DB, Decornez H, Furr JR, Bajorath J (2004) Docking and scoring in virtual screening for drug discovery: methods and applications. Nat Rev Drug Discov 3(11):935–949 15520816 10.1038/nrd1549

[5] Trott O, Olson AJ (2010) Autodock vina: improving the speed and accuracy of docking with a new scoring function, efficient optimization, and multithreading. J Comput Chem 31(2):455–461 19499576 10.1002/jcc.21334 PMC3041641

[6] Friesner RA, Banks JL, Murphy RB, Halgren TA, Klicic JJ, Mainz DT, Repasky MP, Knoll EH, Shelley M, Perry JK et al (2004) Glide: a new approach for rapid, accurate docking and scoring. 1. Method and assessment of docking accuracy. J Med Chem 47(7):1739–1749 15027865 10.1021/jm0306430

[7] Halgren TA, Murphy RB, Friesner RA, Beard HS, Frye LL, Pollard WT, Banks JL (2004) Glide: a new approach for rapid, accurate docking and scoring. 2. Enrichment factors in database screening. J Med Chem 47(7):1750–1759 15027866 10.1021/jm030644s

[8] McNutt AT, Francoeur P, Aggarwal R, Masuda T, Meli R, Ragoza M, Sunseri J, Koes DR (2021) Gnina 1.0: molecular docking with deep learning. J Cheminform 13(1):1–20 34108002 10.1186/s13321-021-00522-2 PMC8191141

[9] Corso G, Stärk H, Jing B, Barzilay R, Jaakkola, T (2023) Diffdock: diffusion steps, twists, and turns for molecular docking. In: International conference on learning representations (ICLR)

[10] Vidal D, Garcia-Serna R, Mestres J (2011) Ligand-based approaches to in silico pharmacology. Chemoinform Comput Chem Biol. 10.1007/978-1-60761-839-3_19 10.1007/978-1-60761-839-3_19 20838981

[11] Banegas-Luna A-J, Ceron-Carrasco JP, Perez-Sanchez H (2018) A review of ligand-based virtual screening web tools and screening algorithms in large molecular databases in the age of big data. Future Med Chem 10(22):2641–2658 30499744 10.4155/fmc-2018-0076

[12] Grimm M, Liu Y, Yang X, Bu C, Xiao Z, Cao Y (2020) Ligmate: a multifeature integration algorithm for ligand-similarity-based virtual screening. J Chem Inf Model 60(12):6044–6053 33190499 10.1021/acs.jcim.9b01210

[13] Broccatelli F, Brown N (2014) Best of both worlds: on the complementarity of ligand-based and structure-based virtual screening. J Chem Inf Model 54(6):1634–1641 24877883 10.1021/ci5001604 PMC4068864

[14] Kumar A, Zhang KY (2018) A cross docking pipeline for improving pose prediction and virtual screening performance. J Comput Aided Mol Des 32:163–173 28836076 10.1007/s10822-017-0048-z

[15] Liu J, Su M, Liu Z, Li J, Li Y, Wang R (2017) Enhance the performance of current scoring functions with the aid of 3d protein-ligand interaction fingerprints. BMC Bioinform 18(1):1–22 10.1186/s12859-017-1750-5 PMC5516336 28720122

[16] Lam PC-H, Abagyan R, Totrov M (2018) Ligand-biased ensemble receptor docking (LigBEnD): a hybrid ligand/receptor structure-based approach. J Comput Aided Mol Des 32:187–198 28887659 10.1007/s10822-017-0058-x PMC5767200

[17] Huang S-Y, Li M, Wang J, Pan Y (2016) Hybriddock: a hybrid protein-ligand docking protocol integrating protein-and ligand-based approaches. J Chem Inf Model 56(6):1078–1087 26317502 10.1021/acs.jcim.5b00275

[18] Paggi JM, Belk JA, Hollingsworth SA, Villanueva N, Powers AS, Clark MJ, Chemparathy AG, Tynan JE, Lau TK, Sunahara RK et al (2021) Leveraging nonstructural data to predict structures and affinities of protein-ligand complexes. Proc Natl Acad Sci 118(51):2112621118 10.1073/pnas.2112621118 PMC8713799 34921117

[19] Bakan A, Meireles LM, Bahar I (2011) Prody: protein dynamics inferred from theory and experiments. Bioinformatics 27(11):1575–1577 21471012 10.1093/bioinformatics/btr168 PMC3102222

[20] Zhang S, Krieger JM, Zhang Y, Kaya C, Kaynak B, Mikulska-Ruminska K, Doruker P, Li H, Bahar I (2021) Prody 2.0: increased scale and scope after 10 years of protein dynamics modelling with python. Bioinformatics 37(20):3657–3659 33822884 10.1093/bioinformatics/btab187 PMC8545336

[21] Berman HM, Westbrook J, Feng Z, Gilliland G, Bhat TN, Weissig H, Shindyalov IN, Bourne PE (2000) The protein data bank. Nucleic Acids Res 28(1):235–242 10592235 10.1093/nar/28.1.235 PMC102472

[22] Burley SK, Bhikadiya C, Bi C, Bittrich S, Chao H, Chen L, Craig PA, Crichlow GV, Dalenberg K, Duarte JM et al (2023) RCSB protein data bank (rcsb.org): delivery of experimentally-determined PDB structures alongside one million computed structure models of proteins from artificial intelligence/machine learning. Nucleic Acids Res 51(D1):488–508 36420884 10.1093/nar/gkac1077 PMC9825554

[23] Schrödinger, LLC: the PyMOL molecular graphics system, version 1.8 (2015)

[24] Riniker S, Landrum GA (2015) Better informed distance geometry: using what we know to improve conformation generation. J Chem Inf Model 55(12):2562–2574 26575315 10.1021/acs.jcim.5b00654

[25] Wang S, Witek J, Landrum GA, Riniker S (2020) Improving conformer generation for small rings and macrocycles based on distance geometry and experimental torsional-angle preferences. J Chem Inf Model 60(4):2044–2058 32155061 10.1021/acs.jcim.0c00025

[26] Francoeur PG, Masuda T, Sunseri J, Jia A, Iovanisci RB, Snyder I, Koes DR (2020) Three-dimensional convolutional neural networks and a cross-docked data set for structure-based drug design. J Chem Inf Model 60(9):4200–4215 32865404 10.1021/acs.jcim.0c00411 PMC8902699

[27] Sunseri J, Koes DR (2016) Pharmit: interactive exploration of chemical space. Nucleic Acids Res 44(W1):442–448 10.1093/nar/gkw287 PMC4987880 27095195

[28] Rohatgi A (2022) Webplotdigitizer: version 4.6. https://automeris.io/WebPlotDigitizer

