# Supplementary material for: Open-ComBind: harnessing unlabeled data for improved binding pose prediction
Source: J Comput Aided Mol Des. 2023 Dec 8;38(1):3. doi: 10.1007/s10822-023-00544-y (PMC10703974; doi:10.1007/s10822-023-00544-y)
Supplement: Supplementary file 1 — Supplementary file1 (PDF 512 kb) [file 10822_2023_544_MOESM1_ESM.pdf]

Supporting Information:
Open-ComBind: Harnessing unlabeled data for improved binding pose prediction

Andrew T. McNutt and David Ryan Koes

Department of Computational and Systems Biology, University of Pittsburgh, Pittsburgh, PA.

Contributing authors: and.mcnutt@pitt.edu; dkoes@pitt.edu;

Protein Name	PDB ID of docking structure	Avg RMSD of pocket	# of docking ligands	# of docking ligands w/ ≤ 2 Å RMSD pose
5HT2B	4IB4	0.524	5	5
AR	2AXA	0.351	19	12
B1AR	2VT4	0.388	11	6
B2AR	2RH1	0.729	7	4
BACE1	1FKN	0.413	20	10-12
BRD4	2YEL	0.320	16	9-10
CDK2	1AQ1	0.956	20	19-20
DAT	4M48	0.409	8	7
DHFR	1BOZ	0.269	20	14,15,18
ELANE	3Q77	0.210	8	0
ERA	1A52	0.513	20	12-13
F10	1EZQ	0.344	20	15-17
F2	1A4W	0.355	20	14-15
GLUT1	5EQG	0.352	2	0
HSP90AA1	1YC1	0.356	20	11-12
MGLUR5	4OO9	0.326	4	2
NR3C1	3BQD	0.705	16	9-10
NR3C2	3WFF	0.421	12	10
P00760	1BJU	0.217	20	13-14
P19491	1FTM	0.607	15	6-7
P22756	1VSO	1.124	18	13-14
PDE10A	3UI7	0.408	20	18
PLAU	1C5X	0.244	20	13
PTPN1	1BZC	0.403	20	11-12
PYGM	1A8I	0.232	20	4
Q05586-Q12879	5H8H	0.332	8	5
SIGMAR1	5HK1	0.295	4	3-4
SLC6A4	5I6X	0.459	4	4
SMO	4JKV	0.591	4	2
VDR	1DB1	0.213	20	3

Table S1: Information about the receptors and ligands in the benchmark dataset. PDB IDs of the crystal structures used for aligning, to define the ground truth location of the cross-docked ligand, and for docking, with the cognate ligand used to define the binding box. Average RMSD of the pocket residues closest to the cognate ligand of the docking structure compared to the docking structure pocket residues. The number of docking ligands for each receptor and the number of ligands where GNINA has sampled a ≤ 2 Å RMSD (multiple numbers provided when different docking seeds had different numbers of ligands).

Parameter	Value
`exhaustiveness`	16
`min_rmsd_filter`	0.01
`num_modes`	30

Table S2: Non-default GNINA parameters used for cross-docking

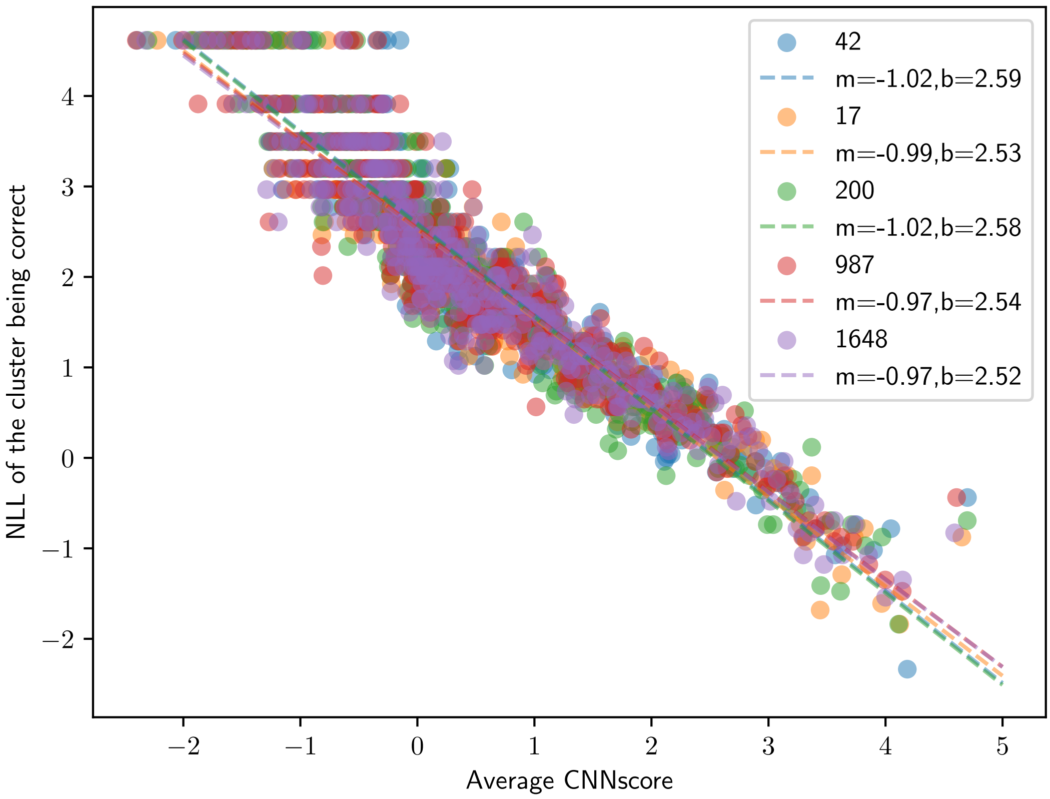

Fig. S1: Hyperparameter C is selected by using the slope of the best fit line between the average CNNscore and negative log likelihood of a cluster of 100 poses being correct. We set C to -1 for simplicity as the slopes of all the lines are nearly -1.

Interaction	Mean	Standard Deviation
Hydrogen bond	127644.6	576.61
Salt bridge	13510.4	48.754
Hydrophobic Contacts	848482.6	507.60

Table S3: Average number of intramolecular interactions seen across the entire benchmark dataset for each of the random seeds used.

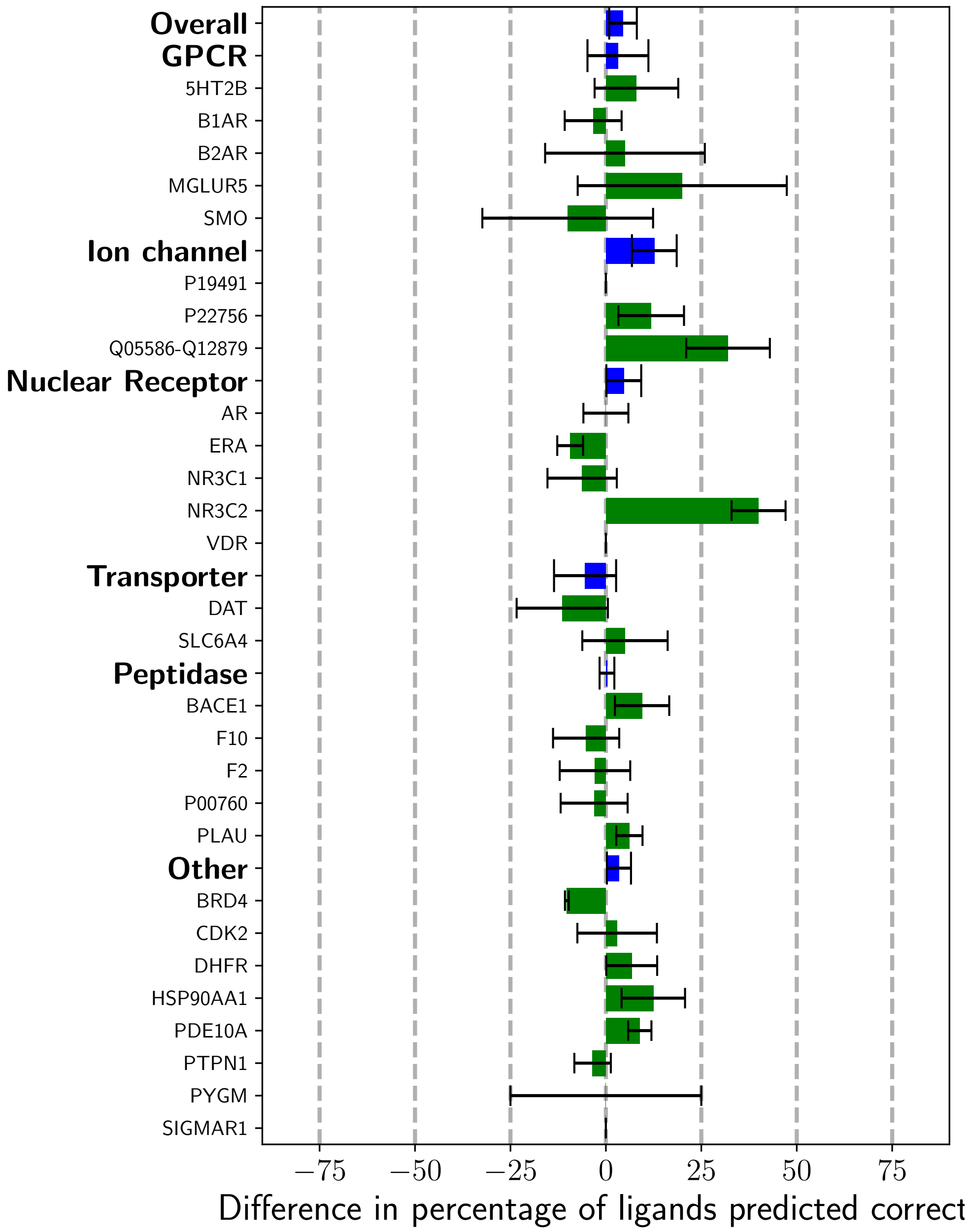

Fig. S2: **Congeneric series helper ligands**: Average difference in percent of ligands whose pose is predicted correctly between GNINA's top scoring pose and the pose selected by Open-ComBind when highly similar ligands are used as helpers. Errorbars show the standard deviation across the five seeds.

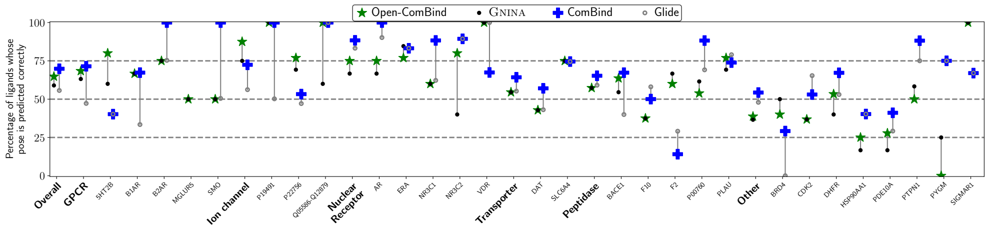

Fig. S3: **Congeneric series helper ligands**:Comparing the docking performance of Open-ComBind and GNINA to the performance of ComBind and Glide when utilizing highly similar ligands as helpers. Performance of ComBind and Glide scraped using WebPlotDigitizer.

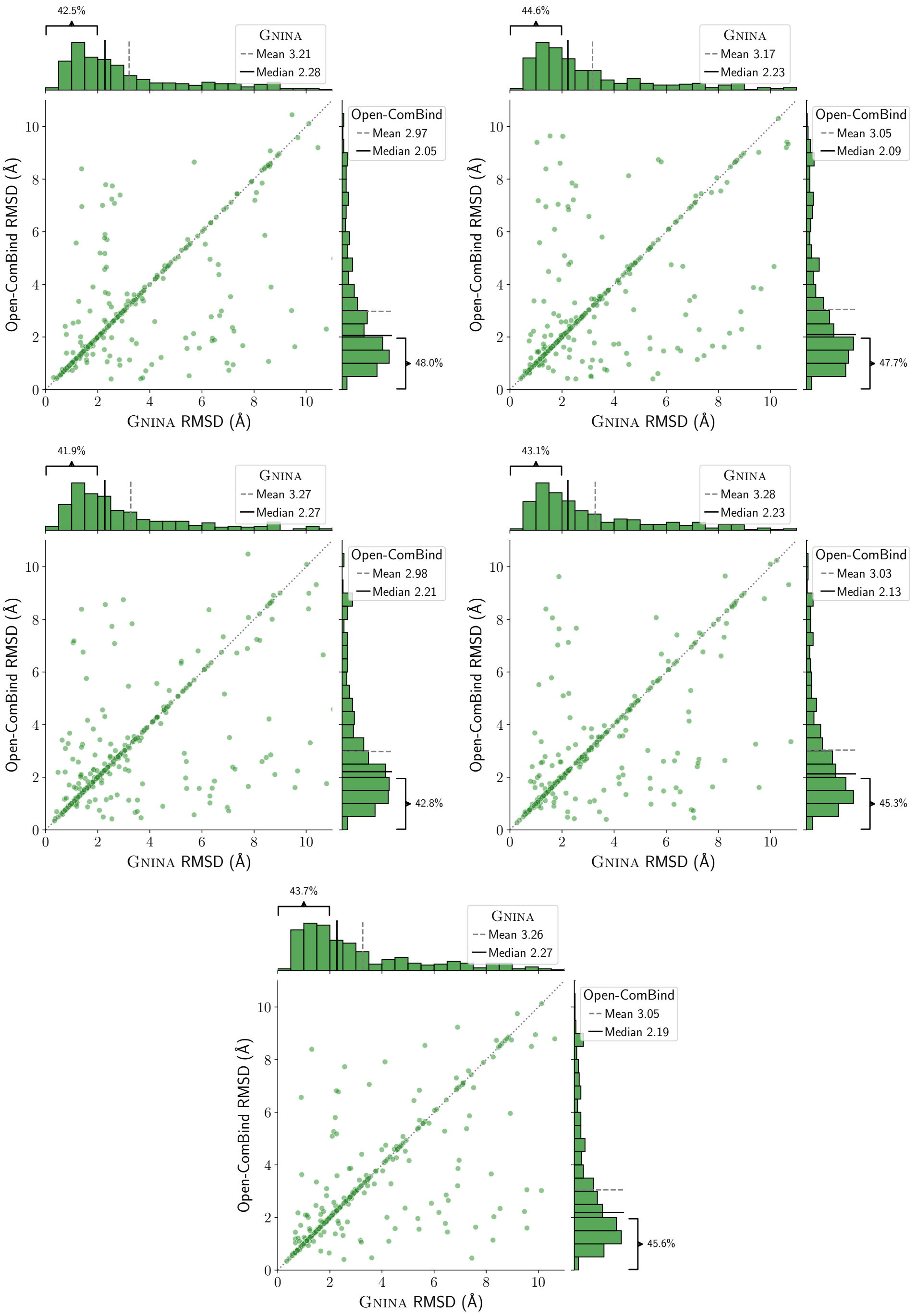

Fig. S4: **High affinity helper ligands**: RMSD to the ground truth of the top pose selected by GNINA and the pose selected by Open-ComBind when the helper ligands are dissimilar to the docking ligand. Brackets indicate the percentage of ligands whose RMSD from the ground truth is ≤ 2 Å. Each graph depicts the results when using one of the five random seeds that are used for ligand conformation generation and docking.

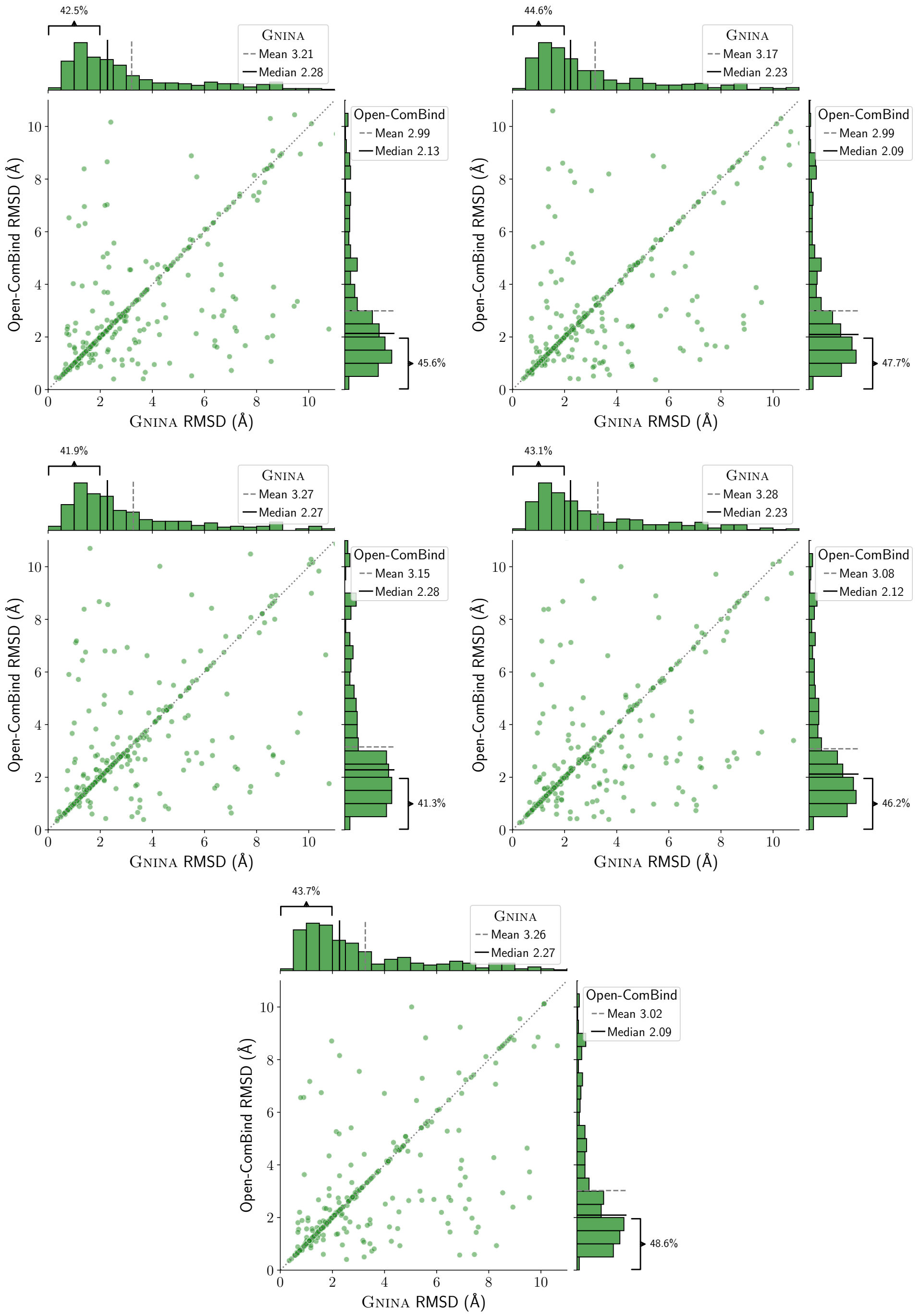

Fig. S5: **Congeneric series helper ligands**: RMSD of the top pose selected by GNINA and the pose selected by Open-ComBind when the helper ligands are highly similar to the docking ligand. Brackets indicate the percentage of ligands whose RMSD from the ground truth is ≤ 2 Å. Each graph depicts the results when using one of the five random seeds that are used for ligand conformation generation and docking.

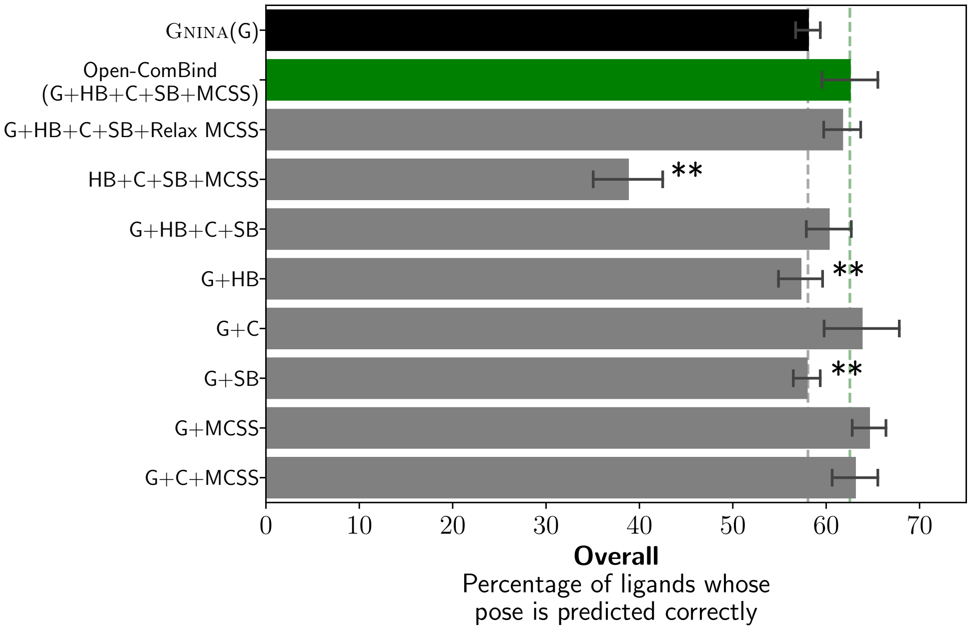

Fig. S6: **Congeneric series helper ligands**: Removing components of Open-ComBind to determine their importance to the selection of docked poses. 'G' indicates that GNINA scores are used, 'HB','C', and 'SB' indicate that hydrogen-bond, hydrophobic contact, and saltbridge similarity is used, respectively. 'MCSS' indicates that the RMSD between the MCSS of ligand poses is used and 'Relax MCSS' indicates a relaxed MCSS strategy was used where strict atom and bond typing were not enforced. '$\cdot$' indicates a p-value less than 0.05 while '$**$' denotes a p-value less than 0.01 in comparison to standard Open-ComBind.

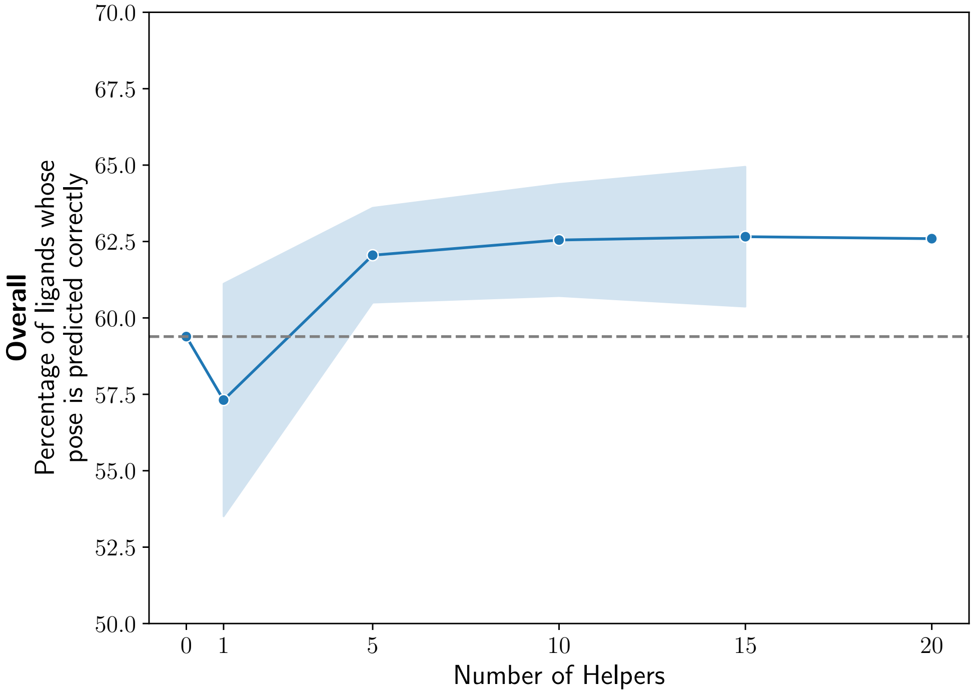

Fig. S7: **Congeneric series helper ligands**: Reducing the number of dissimilar helper ligands used in the Open-ComBind pipeline decreases the number of docked ligands whose pose is predicted correctly. The line is the average and the shaded area is the standard deviation across 15 different subsamples of helper ligands for each number of helper ligands.

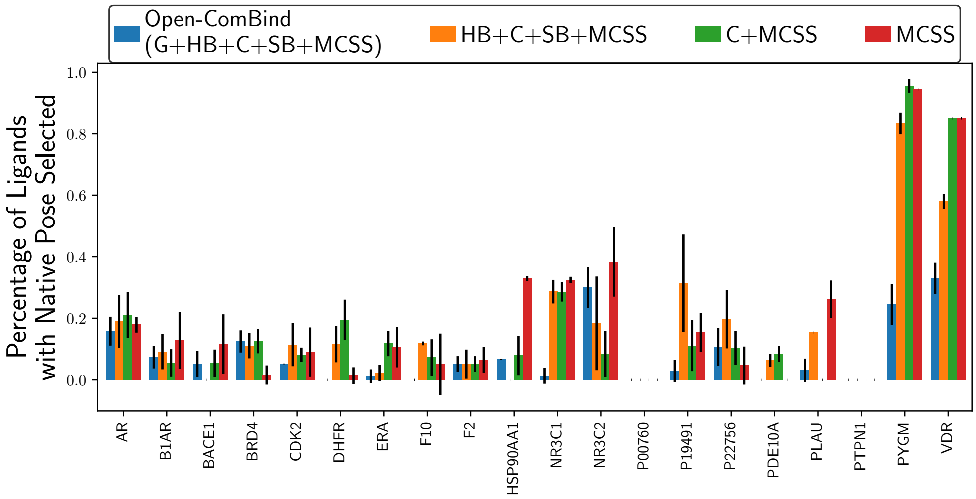

Fig. S8: The native pose of the ligands is included in the pose list during pose selection. The percentage of ligands where the native pose is chosen for varying featurizations of the Open-ComBind pipeline.
